# Prevalence Trends and Patterns of Perinatal ADHD Stimulant Medication Use in British Columbia, Canada

**DOI:** 10.1002/pds.70129

**Published:** 2025-03-23

**Authors:** Amanda Nitchske, Ali Salmanpour, Paramdeep Kaur, Helena Abreu do Valle, Chelsea Elwood, Anne Gadermann, Martin Guhn, Catriona Hippman, Angie Ip, Jalisa L. Karim, Tim F. Oberlander, Peter M. Socha, Gillian E. Hanley

**Affiliations:** ^1^ Department of Obstetrics & Gynaecology University of British Columbia Vancouver British Columbia Canada; ^2^ BC Children's Hospital Research Institute University of British Columbia Vancouver British Columbia Canada; ^3^ School of Population and Public Health University of British Columbia Vancouver British Columbia Canada; ^4^ Human Early Learning Partnership University of British Columbia Vancouver British Columbia Canada; ^5^ BC Reproductive Mental Health Program BC Women's Hospital & Health Centre Vancouver British Columbia Canada; ^6^ Women's Health Research Institute Vancouver British Columbia Canada; ^7^ Division of Developmental Pediatrics, Department of Pediatrics University of British Columbia Vancouver British Columbia Canada; ^8^ Sunny Hill Health Centre BC Children's Hospital Vancouver British Columbia Canada; ^9^ Department of Epidemiology, Biostatistics and Occupational Health McGill University Quebec Canada

**Keywords:** attention deficit/hyperactivity disorder (ADHD), drug utilization study, pregnancy, stimulants

## Abstract

**Purpose:**

Given the increase in attention‐deficit/hyperactivity disorder (ADHD) diagnoses and stimulant medication use among female adults, this study describes the prevalence trends of perinatal ADHD stimulant medication use in British Columbia, Canada, along with characteristics and patterns of use.

**Methods:**

Using linked population‐based administrative data, we included all pregnant people with deliveries between January 2000 and December 2021. ADHD stimulant medication use was defined as filled prescriptions for dextro−/amphetamine, methylphenidate, or lisdexamfetamine. Prevalence trends were examined by medication type and age group. Characteristics were compared between those with and without prenatal stimulant medication dispensations. Patterns of use and discontinuation were assessed from 1 year preconception to 1 year postpartum.

**Results:**

Our cohort included 899,679 pregnancies. Prenatal ADHD stimulant medication use increased by 3.9 users per 1000 pregnancies (from 0.4 to 4.3/1000), primarily driven by dextro−/amphetamine. Medication use increased among all age groups, but was highest among pregnant people under 20 years old. Pregnant people taking stimulant medications were more likely to be nulliparous and lower in income, have hypertension and higher BMI, smoke during pregnancy, use other psychotropic medications, and deliver by cesarean section. Among those who used stimulant medications within 1 year preconception, 77% discontinued treatment before or during pregnancy. While use increased again within 12 months postpartum, it remained 45% lower than preconception levels.

**Conclusion:**

The 11‐fold increase in ADHD stimulant medication use during pregnancy and the high rate of discontinuation highlight the need for more research on the risks and benefits of medication for parent and child health.

**Summary**

- There was an 11‐fold increase in ADHD stimulant medication use during pregnancy between 2000 and 2021 in British Columbia, Canada.
- Dextro−/amphetamine and methylphenidate were the most common therapeutic choices, with lisdexamfetamine dispensations becoming more common from 2015 onward.
- Prenatal ADHD stimulant medication use was associated with several sociodemographic and pregnancy complications and risk factors, including lower income, smoking, hypertension, and the use of other psychotropic medications; therefore, it is crucial that future studies assessing the safety of ADHD stimulant medication during pregnancy are methodologically rigorous to adequately address these confounding factors.

## 1 Introduction

Attention‐deficit hyperactivity disorder (ADHD) is a neurodevelopmental condition that affects attention, impulse control, planning, organization, emotional reactivity, and mood [1]. ADHD typically presents in childhood, with a prevalence between 3% and 7%, and symptoms persist into adulthood in approximately 60%–75% of individuals [1, 2, 3].

Over the past decade, the incidence and prevalence of adult ADHD diagnoses have risen, especially among adult females, with 3.2% of adult women and 4.3% of gender‐diverse adults assigned female at birth meeting the criteria for ADHD [4, 5]. This increase in female ADHD diagnoses may reflect a correction to historical sex and gender biases in ADHD diagnostic criteria. The presentation of ADHD also differs by sex and gender, with girls being more likely to present with inattentive‐type ADHD, which is less likely to be perceived as disruptive [6]. The new Diagnostic and Statistical Manual of Mental Disorders, Fifth Edition (DSM‐5) criteria for diagnosing ADHD appear more aligned with the presentation of ADHD in females than DSM‐IV criteria, which may reflect that DSM‐5 field studies included more females than DSM‐IV [6, 7, 8].

The standard treatment for ADHD consists of a combination of behavioral therapy and pharmacological treatments [9]. Meta‐analyses of double‐blind RCTs have found that stimulant ADHD medications are highly and non‐stimulant medications moderately efficacious in reducing the core symptoms of ADHD [10]. As a result, the first‐line therapeutic choice to treat ADHD is stimulant medications, which exert their effects by increasing levels of the neurotransmitters dopamine and norepinephrine in the brain. Nonstimulant medications are a second‐line treatment option for the minority of cases in which stimulants do not work or individuals experience severe side effects, such as cardiovascular, growth, or neuropsychiatric complications [11, 12].

Recent estimates put the prevalence of stimulant medication use at 5.6% of young adults in Canada—a rate that has been increasing in recent years [13]. In addition, recent research shows that the prescription prevalence of ADHD medication has increased most rapidly among adult females of childbearing ages, raising critical and yet under‐investigated questions about the developmental effects of fetal stimulant exposure [14, 15, 16]. Several countries have reported significant increases in ADHD medication use specifically during the perinatal period, with 6.5‐ to 14‐fold increases reported in North America [17, 18] and 2‐ to 106‐fold increases in Europe [19, 20]. This variation in estimates is likely influenced by differences in the time period studied, medications included, and defined exposure period of interest. Overall, it is crucial to examine up‐to‐date trends and determinants of ADHD medication use among pregnant people in order to help guide further research and to ultimately promote informed clinical decision‐making regarding treatment during pregnancy.

In light of these growing international prevalence trends in female adult ADHD diagnosis and stimulant medication use, we hypothesized that ADHD medication use during pregnancy may have also increased in British Columbia (BC), Canada. Thus, this study aimed to describe the prevalence trends in perinatal ADHD stimulant medication use and compare characteristics of: (a) Pregnant people who used ADHD stimulant medication versus pregnant people with no use, and (b) pregnant people who used ADHD stimulant medication versus pregnant people who discontinued these medications during pregnancy but used them within 1 year before pregnancy (i.e., continuers vs. discontinuers). Throughout this paper, the term perinatal is used to encompass the period from one year preconception to one year postpartum.

## 2 Methods

### 2.1 Study Cohort and Data Sources

This study used population‐based administrative data from BC, Canada. The cohort included all pregnant people with deliveries between January 1st 2000 and December 31st 2021, in BC. Population Data BC linked data from the BC Perinatal Data Registry (BCPDR) [21], the BC PharmaNet, and the BC Consolidation file. The BCPDR is a province‐wide registry that contains information on antenatal, intrapartum, and postpartum care and outcomes of birthing parents and infants for over 99% of deliveries in BC, including home births. These data were used to build our cohort and include live births and stillbirths (defined as fetal death occurring after at least 20 weeks of gestation, or achieving a weight of at least 500 g). In addition, the BCPDR provides an algorithm‐based estimate of gestational age at birth, which is based on information from early gestational ultrasound (< 20 weeks), the date of the last menstrual period (LMP) (if there was no early gestational ultrasound), a clinical estimate from a newborn exam if both early ultrasound and LMP are missing, and documentation from the birth parent's chart if all three prior data sources are missing. This information was used to define a pregnancy period and differentiate between pregnancy trimesters with as much precision as possible. These data were linked with the BC PharmaNet, which contains all prescriptions dispensed in an outpatient setting in BC, and the BC Consolidation file, which provides demographic and census geocodes data.

Further information regarding these data sets can be found on Population Data BC's project webpage (https://my.popdata.bc.ca/project_listings/20‐167/collection_approval_dates). Access to data provided by the Data Stewards is subject to approval but can be requested for research projects through the Data Stewards or their designated service providers. All inferences, opinions, and conclusions drawn in this publication are those of the author(s), and do not reflect the opinions or policies of the Data Steward(s).

Ethics approval was obtained from the UBC Clinical Research Ethics Board. Approval by the Ethics Board and the BC Data stewards for the use of deidentified administrative data files includes a waiver of informed consent from participants.

### 2.2 Exposure

Prenatal ADHD stimulant medication exposure was defined as having filled one or more prescriptions for dextro−/amphetamine, methylphenidate, or lisdexamfetamine from the date of conception to the date of delivery. The date of conception was estimated by subtracting two weeks from the baby's final gestational age at birth (to reflect the two weeks of gestational age that reflect time prior to conception) and then subtracting these weeks from the baby's date of birth. To assess exposure timing, we categorized medication use by trimester based on the dispensation date and the number of days supplied. Additionally, we identified everyone who had at least one ADHD stimulant medication dispensation from 1 year preconception to 1 year postpartum. Exposure status was ascertained using the BC PharmaNet data.

### 2.3 Statistical Analysis

Prevalence trends in ADHD stimulant medication use during pregnancy from 2000 to 2021 were examined overall, by type of medication, and by age group. Characteristics of those filling prenatal ADHD stimulant medication prescriptions were compared to those not filling these prescriptions using standardized mean differences (SMD). Based on previous guidelines, we considered a characteristic to have a meaningful difference between the exposure groups if the SMD was greater than 0.1 [22]. Characteristics included: age at delivery, income quintile during the year of delivery, parity, smoking during pregnancy, preexisting diabetes, gestational diabetes, pregnancy‐induced hypertension and other types of hypertension (which includes individuals with preexisting hypertension [blood pressure ≥ 140/90], hypertension associated with chronic renal disease in pregnancy, hypertension as a result of another cause during pregnancy, labour, or postpartum period, or having received antihypertensive drugs during pregnancy), pre‐pregnancy body mass index (kg/m^2^), other prenatal psychotropic medication (antidepressants, antipsychotics and anxiolytics, as mentioned in Table S1), mode of delivery, and calendar year of delivery.

In addition, we calculated the prevalence of use 1 year and 6 months before the calculated date of conception as well as 6 months and 1 year postpartum. We examined differences between people who continued versus discontinued their ADHD pharmacotherapy during pregnancy by restricting our main cohort to a sub‐cohort of people who had ADHD stimulant medication dispensations during the one year before their date of conception. Within this sub‐cohort, we compared characteristics among those who continued treatment throughout their entire pregnancy (i.e., continuers) to those who discontinued treatment before or during pregnancy (1st, 2nd, or 3rd trimester discontinuation). For example, those who filled at least one prescription during 1 year preconception but did not fill any prescriptions during their 1st, 2nd, and 3rd trimesters were considered 1st trimester discontinuers. Those who filled prescriptions during 1 year preconception and their 1st trimester but not their 2nd or 3rd trimester were considered 2nd trimester discontinuers, etc. The statistical analysis was performed using RStudio software (R version 4.0.5).

## 3 Results

**FIGURE 1:**
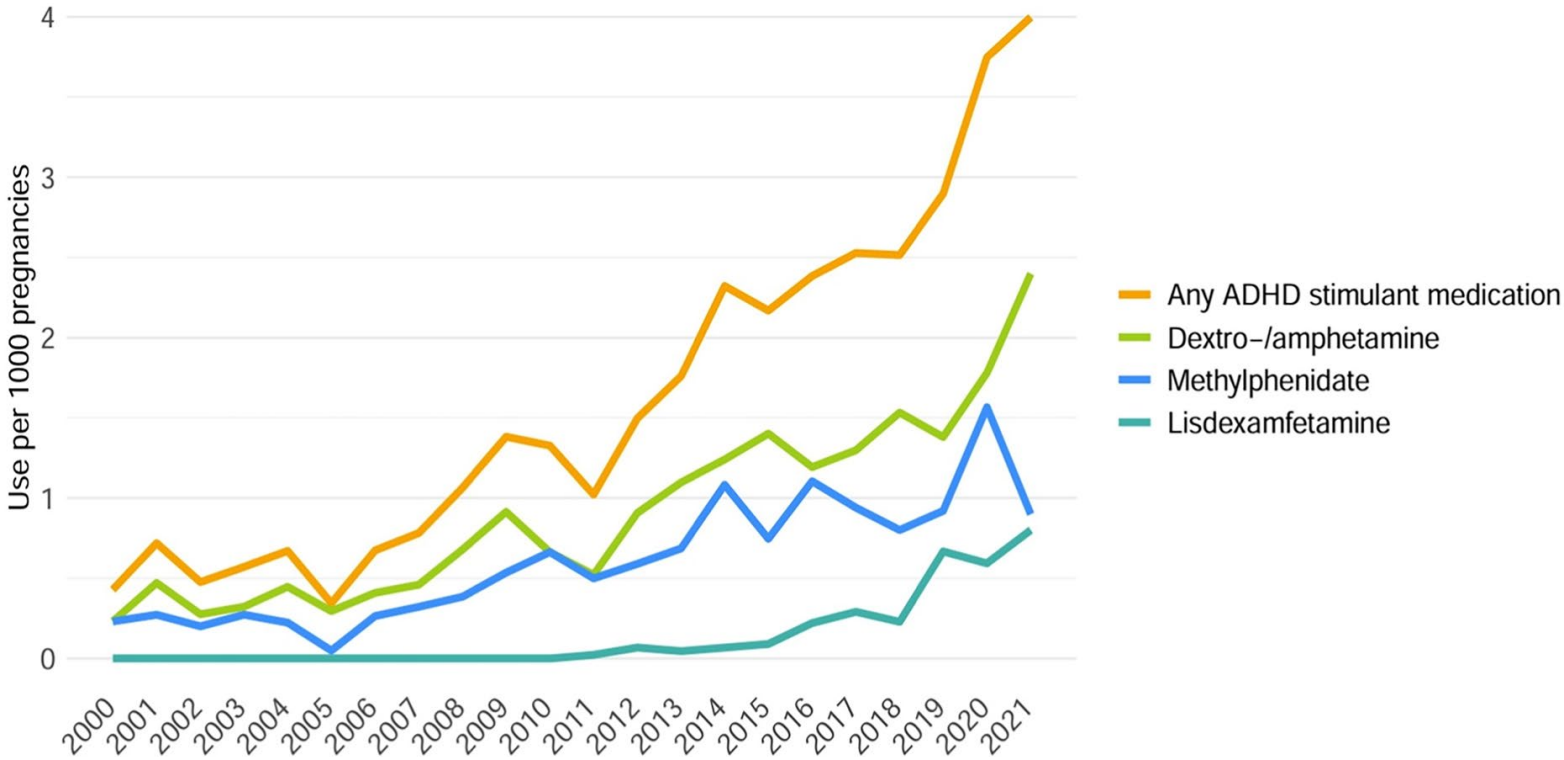
Prevalence of ADHD stimulant medication use during pregnancy from 2000 to 2021 overall and by specific medication in British Columbia, Canada.

**FIGURE 2:**
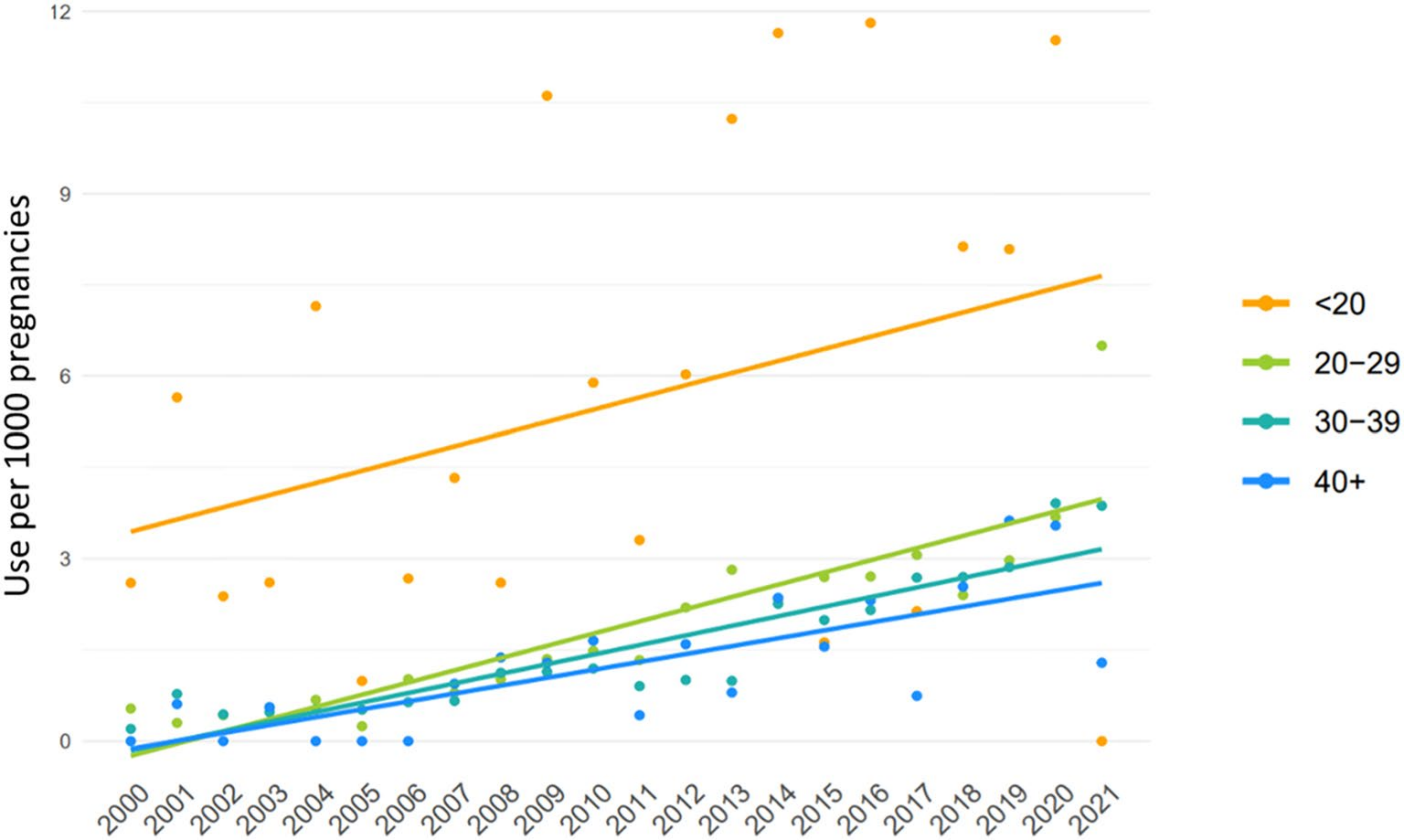
Prevalence of ADHD stimulant medication use (per 1000 pregnancies) from 2000 to 2021, categorized by age group. Individual data points are shown for each year, and a linear regression line is fitted to illustrate trends in ADHD stimulant medication use over time for each age group. The lines represent estimated trends based on a linear model.

There were 899,679 pregnancies to 543,735 people between January 1st 2000, and December 31st 2021, of which 1396 (0.16%) pregnancies were exposed to ADHD stimulant medication at any point between the date of conception and the date of delivery. Prenatal ADHD stimulant medication use increased by 3.9 users per 1000 pregnancies over our study period (from 0.4 in 2000 to 4.3/1000 in 2021), representing an 11‐fold increase. This increase was primarily driven by dextro−/amphetamine, followed by methylphenidate, and from 2015 onward, lisdexamfetamine (Figure 1). Further, while prenatal ADHD stimulant medication use increased among all age groups, it was highest among pregnant people under 20 years of age (Figure 2).

There were important differences between pregnant people exposed and unexposed to prenatal ADHD stimulants (Table 1). Pregnant people taking ADHD stimulant medications were younger (< 20 years, users: 6.6% vs. non‐users: 2.1%), more likely to be in lower income quintiles (lowest income quintile, users: 22.3% vs. non‐users: 16.6%), more commonly nulliparous (52.0% vs. 46.2%), and were more likely to have smoked during pregnancy (21.8% vs. 8.4%), be higher in BMI (≥ 30.0, users: 13.9% vs. 9.5%), have hypertension (pregnancy‐induced: 9.4% vs. 5.4%, and other hypertension: 6.2% vs. 3.1%), use other psychotropic medications (antidepressants: 34.1% vs. 4.3%, antipsychotics: 9.9% vs. 0.6%, and anxiolytics: 13.5% vs. 2.5%), and deliver by cesarean section (38.8% vs. 32.0%).

**TABLE 1:** Characteristics of ADHD stimulant medication users during pregnancy compared to non‐users during pregnancy in the full cohort (*N* = 899,679).

	Prenatal ADHD stimulant medication		Standardized mean difference
	No (*N* = 898 283)	Yes (*N* = 1396)	
Age at delivery (years), mean (SD)	30.94 (5.49)	29.98 (6.16)	0.165
Age at delivery categories (years), No. (%)			0.229
< 20	18 537 (2.1)	93 (6.6)	
20–29	325 585 (36.2)	501 (35.9)	
30–39	506 095 (56.3)	735 (52.7)	
40+	48 066 (5.4)	67 (4.8)	
Income quintile during year of delivery, No. (%)			0.168
1 (lowest)	134 502 (16.6)	219 (22.3)	
2	149 086 (18.4)	259 (19.9)	
3	164 841 (20.3)	248 (19.0)	
4	178 013 (20.3)	256 (19.6)	
5 (highest)	185 244 (22.8)	249 (19.1)	
Missing	86 597 (9.6)	93 (6.7)	
Parity, No. (%)			0.116
Nulliparous	415 126 (46.2)	726 (52.0)	
Multiparous	483 123 (53.8)	670 (48.0)	
Smoked during pregnancy, No. (%)	75 154 (8.4)	304 (21.8)	0.382
Diabetes, No. (%)			
Pre‐existing	4757 (0.5)	17 (1.2)	0.074
Gestational	86 088 (9.6)	142 (10.2)	0.020
Hypertension, No. (%)			
Pregnancy‐induced	48 125 (5.4)	131 (9.4)	0.155
Other^a^	27 901 (3.1)	86 (6.2)	0.146
Pre‐pregnancy body mass index (kg/m^2^), No. (%)			0.155
< 18.5 (underweight)	39 634 (4.4)	47 (3.5)	
18.5–24.9 (normal)	395 187 (44.0)	570 (40.8)	
25.0–29.9 (overweight)	138 221 (15.4)	236 (16.9)	
≥30.0 (obese)	85 674 (9.5)	194 (13.9)	
Missing	239 567 (26.7)	349 (25.0)	
Other prenatal psychotropic medication use, No. (%)			
Antidepressants	38 542 (4.3)	476 (34.1)	0.818
Antipsychotics	5592 (0.6)	138 (9.9)	0.424
Anxiolytics	22 266 (2.5)	188 (13.5)	0.414
Mode of delivery, No. (%)			0.143
Cesarian section	287 017 (32.0)	541 (38.8)	
Vaginal delivery	611 266 (68.0)	855 (61.2)	
Year of delivery, No. (%)			0.594
2000–2005	231 704 (25.8)	125 (9.0)	
2006–2010	217 635 (24.2)	229 (16.4)	
2011–2015	220 195 (24.5)	387 (27.7)	
2016–2021	228 749 (25.5)	655 (46.9)	

^a^ Consists of preexisting hypertension (blood pressure ≥ 140/90), received antihypertensive drugs during pregnancy, hypertension associated with chronic renal disease in pregnancy, and hypertension as a result of another cause during pregnancy, labour, or postpartum period.

### 3.1 Continuer Versus Discontinuers

**FIGURE 3:**
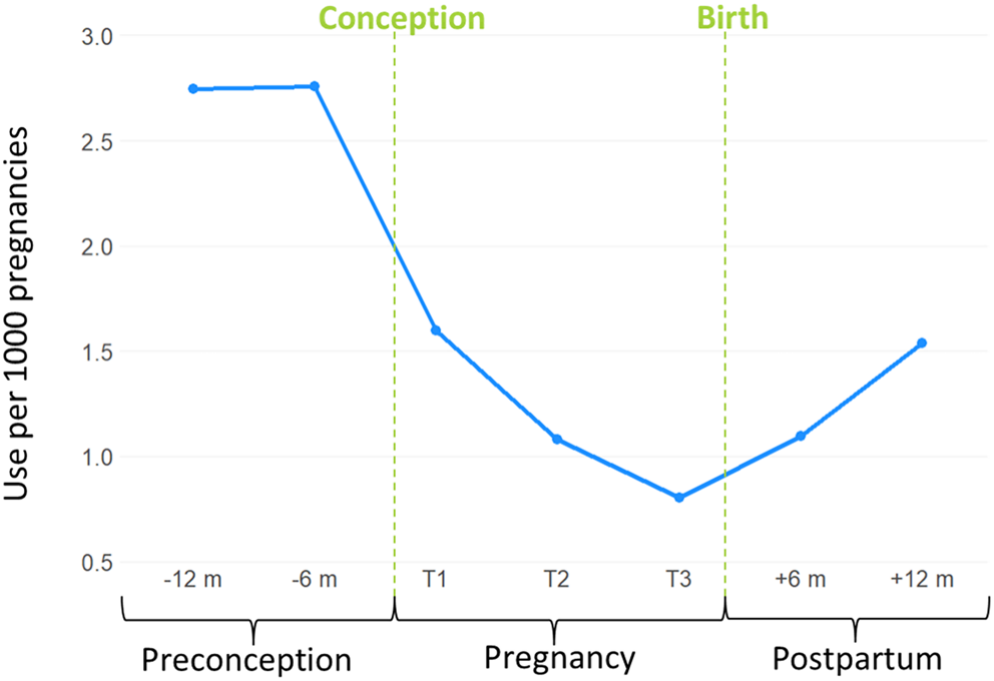
Prevalence of ADHD stimulant medication use from 1 year preconception to 1 year postpartum in British Columbia, Canada.

Among those who used ADHD stimulant medications within 1 year preconception, 77% discontinued treatment before or during pregnancy. More specifically, 54.8% (1588/2895) discontinued their medication before their first trimester (before conception), 14.3% (414/2895) discontinued before their second trimester, and 7.9% discontinued before their third trimester (228/2895) (Table 2). Upon comparing characteristics between those who continued ADHD stimulant medication treatment throughout pregnancy versus those who discontinued treatment before or during pregnancy, continuers were older, more commonly multiparous, and more likely to have smoked during pregnancy, had hypertension, used other prenatal psychotropic medications, and delivered by cesarean section (Table 3). ADHD stimulant medication use increased by approximately 55% within 12 months postpartum, though it remained lower than preconception levels, with medication use being 2.76 per 1000 pregnancies 1 year preconception compared to 1.53 per 1000 pregnancies 1 year postpartum (Figure 3).

**TABLE 2:** Description of use of ADHD stimulant medication.

	ADHD stimulant medication use
Specific type of medication, No. (per 1000 pregnancies)	
Dextro−/amphetamine	783 (0.87)
Methylphenidate	544 (0.65)
Lisdexamfetamine	108 (0.12)
Number of medications used in pregnancy, No. (%)	
1	1349 (96.6)
2+	47 (3.37)
Number of medications used during 1 year preconception, No. (%)	
1	2668 (92.2)
2+	227 (7.84)
Number of medications used during 1 year postpartum, No. (%)	
1	1483 (92.4)
2+	122 (7.60)
Pattern of ADHD medication use in pregnancy, No. (%)	
Discontinued before or during pregnancy	2230 (77.0)
1^st^ trimester discontinuation	1588 (54.8)
2^nd^ trimester discontinuation	414 (14.3)
3^rd^ trimester discontinuation	228 (7.9)
Continued entire pregnancy	665 (23.0)

**TABLE 3:** Characteristics of ADHD stimulant medication users among those who continued vs. discontinued treatment before pregnancy or at an point during pregnancy (*N* = 2895).

	Discontinued treatment (*N* = 2230)	Continued treatment (*N* = 665)	Standardized mean difference
Age at delivery (years), mean (SD)	28.74 (6.61)	30.70 (5.70)	0.317
Age at delivery categories (years), No. (%)			0.340
< 20	246 (11.0)	25 (3.8)	
20‐29	886 (39.7)	225 (33.8)	
30‐39	1009 (45.2)	385 (57.9)	
40+	89 (4.0)	30 (4.5)	
Income quintile during year of delivery, No. (%)			0.055
1 (lowest)	460 (22.3)	132 (20.9)	
2	409 (19.8)	133 (20.9)	
3	389 (18.8)	128 (20.3)	
4	410 (19.9)	119 (18.8)	
5 (highest)	397 (19.2)	121 (19.1)	
Missing	165 (7.4)	33 (5.0)	
Parity, No. (%)			0.227
Nulliparous	1360 (61.0)	331 (49.8)	
Multiparous	870 (39.0)	334 (50.2)	
Smoked during pregnancy, No. (%)	449 (20.1)	164 (24.7)	0.109
Diabetes, No. (%)			
Pre‐existing	24 (1.1)	10 (1.5)	0.038
Gestational	186 (8.3)	70 (10.5)	0.075
Hypertension, No. (%)			
Pregnancy‐induced	176 (7.9)	78 (11.7)	0.129
Other^a^	87 (3.9)	52 (7.8)	0.167
Pre‐pregnancy body mass index (kg/m^2^), No. (%)			0.081
< 18.5 (underweight)	76 (3.4)	26 (3.9)	
18.5–24.9 (normal)	887 (39.8)	281 (42.3)	
25.0–29.9 (overweight)	360 (16.1)	112 (16.8)	
≥ 30.0 (obese)	346 (15.5)	89 (13.4)	
Missing	561 (25.2)	157 (23.6)	
Other prenatal psychotropic medication use, No. (%)			
Antidepressants	533 (23.9)	242 (36.4)	0.275
Antipsychotics	140 (6.3)	72 (10.8)	0.163
Anxiolytics	198 (8.9)	92 (13.8)	0.157
Mode of delivery, No. (%)			0.108
Cesarian section	798 (35.8)	273 (41.1)	
Vaginal delivery	1432 (64.2)	392 (58.9)	
Year of delivery, No. (%)			0.201
2000–2005	231 (10.4)	45 (6.8)	
2006–2010	388 (1.4)	96 (14.4)	
2011–2015	659 (29.6)	180 (27.1)	
2016–2021	952 (42.7)	344 (51.7)	

^a^ Consists of preexisting hypertension (blood pressure ≥ 140/90), received antihypertensive drugs during pregnancy, hypertension associated with chronic renal disease in pregnancy, and hypertension as a result of another cause during pregnancy, labour, or postpartum period.

## 4 Discussion

Our findings demonstrate an 11‐fold increase in ADHD stimulant medication use during pregnancy between 2000 and 2021. These findings are consistent with other epidemiological studies. A population‐based study from the province of Quebec reported a 14‐fold increase in the rate of ADHD medication use from 0.08% in 1998 to 1.2% in 2015, as well as a 29‐fold increase in the rate of ADHD diagnosis [18]. Moreover, a multi‐site study from the United States found a 6.5‐fold increase in reported ADHD medication use, from 0.2% of pregnant people in 1997–1998 to 1.3% in 2013 [17]. Data from Norway and Sweden showed 2‐fold and 5‐fold increases, respectively, over a 9‐year period from 2010 to 2019 [19], while a Danish study found a 106‐fold increase in the incidence of pregnancies exposed to ADHD medication (from 5 to 533 per 100 000 person‐years) over a 7‐year period from 2003 to the first quarter of 2010 [20]. Similar to Norway, Sweden, and Denmark, we found that pregnant people less than 20 years of age were much more likely to use ADHD medication during pregnancy. This finding may reflect that rates of unplanned pregnancy and early parenthood are significantly higher among people with ADHD [23, 24]. Alternatively, this pattern could reflect a cohort effect, as younger generations, who are more likely to have been diagnosed with ADHD due to increased awareness and changes in diagnostic practices, also account for a greater proportion of deliveries in more recent years among ADHD medication users, as shown in Table 1. While dextro−/amphetamine medications were the most common in our cohort, lisdexamfetamine use showed a steady increase beginning in 2015. This trend aligns with data from Sweden and the UK [19, 25] and may be attributed to the oral long‐acting prodrug design of lisdexamfetamine, which is believed to improve adherence and reduce the likelihood of misuse or abuse by preventing mechanical tampering [26].

In our cohort, the majority (77%) discontinued treatment before or during pregnancy. While medication use began increasing again within 12 months postpartum, it remained approximately 45% lower than preconception levels. This sustained reduction may reflect ongoing concerns about infant exposure during breastfeeding. Studies from Norway, Sweden, and Denmark report similar discontinuation rates: 85% in Norway, 78% in Sweden, and 60% in Denmark [19, 27]. However, these studies also found that the continuation of treatment throughout pregnancy became more common over time [27, 28]. Furthermore, they observed that those who continued treatment differed in sociodemographic and clinical factors, potentially indicating more severe ADHD, such as more frequent medication switches and co‐prescriptions [27]. In our cohort, prenatal ADHD medication use was also associated with several pregnancy complications and risk factors, including smoking, hypertension, and the use of other psychotropic medications (e.g., antidepressants, anxiolytics, and antipsychotics). This is also in line with other literature showing that ADHD commonly coexists with other psychiatric illnesses [29]; around 10% of adults with recurrent depression and/or anxiety disorders have ADHD [30]. Therefore, it is crucial that studies assessing the safety of ADHD medication during pregnancy are methodologically rigorous to adequately address these confounding factors. By providing detailed characteristics of ADHD stimulant medication users during pregnancy, our study may help inform future research by identifying relevant confounders for studies on pregnancy outcomes associated with ADHD medication use.

Psychostimulants are one of the psychotropic classes on which we have the least evidence in the peripartum period, but available studies are reporting generally reassuring safety data [11]. Two systematic reviews of observational data demonstrated that absolute risk differences of various perinatal outcomes were small in magnitude [31, 32]. This was particularly true for studies using alternative comparison groups to rule out confounding, such as comparing exposed and unexposed pregnancies in the same person (i.e., sibling comparison) [20, 31]. Additionally, while small absolute increases in risk for preeclampsia (absolute risk differences [aRD] ranging from 0.39 to 1.13) and preterm birth (aRD ranging from 0.18 to 1.73) remained among those exposed compared to appropriate comparison groups, there is currently no clear evidence to indicate clinically significant adverse effects [31]. While short‐term data on infant outcomes generally look reassuring, very few studies to date have assessed the long‐term developmental impacts of exposure. However, emerging evidence currently shows no long‐term effect on offspring neurodevelopment [33, 34, 35] Long before birth, monoamines, including dopamine and norepinephrine, play critical roles as neurotrophic signals important for placental function and fetal brain development by regulating cell proliferation and neuronal migration [36]. Increasing evidence indicates that even temporary disturbances in monoamine signaling during gestation are capable of inducing sustained changes to the fetal brain structure and functions, extending into adulthood [37]. Thus, the long‐term developmental impacts of prenatal ADHD stimulant medication exposure warrant further investigation.

While the use of any medication during pregnancy has its risks, it is also important to recognize that untreated ADHD during pregnancy carries its own risks [11]. Untreated ADHD has been linked to worse mental health outcomes and significant impairments in functioning in pregnant people, as well as increased risks for spontaneous abortion and preterm birth [11, 38]. Overall, future research should focus on improving our understanding of the risks and benefits of ADHD medication use during pregnancy by adding to the existing evidence base regarding their impact on both birthing parent and child health. This is essential to support informed treatment decision‐making in this understudied population. While exposure to stimulant medications remains low (0.16% of our cohort), the rapidly increasing use of stimulant medications in pregnancy suggests that future work in this area will become increasingly important.

## 5 Strengths and Limitations

This study is strengthened by its longitudinal population‐based cohort that resulted in a large sample size and reduced the potential for selection bias. In addition, the BC Perinatal Data Registry provided validated and detailed information on many prenatal and intrapartum characteristics [39]. This study also has several limitations. First, we were only able to include all pregnancies that resulted in live births or stillbirths delivered after 20 weeks of gestation or achieving a weight of at least 500 g. We were unable to capture pregnancies that ended in miscarriages or abortions, and as a result, ADHD stimulant medication use during pregnancy may be underestimated. This is especially relevant since research suggests that pregnancies in people with ADHD are more likely to end up with these outcomes [20, 40]. We were also unable to determine when blood pressure was measured in relation to the initiation of ADHD stimulant treatment. Since stimulants can elevate blood pressure, some cases of hypertension may have resulted from medication effects. In addition, because clinicians routinely monitor blood pressure in patients prescribed stimulant medications, there is a potential for surveillance bias, leading to increased detection of hypertension in those using ADHD medications. Our dataset lacked information on the severity of ADHD symptoms, which is likely to play an important role in the decision to continue treatment during pregnancy. We also lacked data on dosage for the dispensed stimulant medications. Furthermore, our exposure was defined based on stimulant medication dispensations, which do not necessarily confirm medication use; therefore, there is potential for some exposure misclassification. Finally, this paper focused only on stimulant ADHD medications due to the very low rates of use of other ADHD medications in our cohort (i.e., 39 people filled prescriptions for atomoxetine, 40 for modafinil, and 2 for guanfacine).

## 6 Conclusion

In summary, we found an 11‐fold increase in ADHD stimulant medication use over the past two decades in BC, primarily driven by dextro−/amphetamine. Appropriate treatment of ADHD is an important public health issue, as ADHD is associated with high rates of psychiatric [41, 42] and somatic symptoms and conditions [43, 44], as well as increased risk for poor educational, occupational, and social outcomes [45, 46]. Given the increased use of ADHD stimulant medication during pregnancy and the high rate of discontinuation during pregnancy, more research is needed to improve our understanding of the risks and benefits of prenatal ADHD stimulant medication for parental and child health.

## Ethics Statement

Ethics approval was obtained from the UBC Clinical Research Ethics Board. Approval by the Ethics Board and the BC Data stewards for use of deidentified administrative data files includes a waiver of informed consent from participants.

## Conflicts of Interest

The authors declare no conflicts of interest.

## Supporting information


**Table S1.** Psychotropic medication classification.
