# Supplementary material for: Prevalence Trends and Patterns of Perinatal ADHD Stimulant Medication Use in British Columbia, Canada
Source: Pharmacoepidemiol Drug Saf. 2025 Mar 23;34(4):e70129. doi: 10.1002/pds.70129 (PMC11931092; doi:10.1002/pds.70129)
Supplement: Supplementary file 1 — Table S1. Psychotropic medication classification. [file PDS-34-e70129-s001.docx]

**eTable 1.** **Psychotropic medication classification.**

| Medication | Generic names |
| --- | --- |
| Antidepressants | Selective serotonin reuptake inhibitors (SSRI): Zimeldine, Fluoxetine, Citalopram, Paroxetine, Sertraline, Alaproclate, Fluvoxamine, Etoperidone, Escitalopram  Selective norepinephrine reuptake inhibitors (SNRI): Venlafaxine, Duloxetine, Desvenlafaxine  Non-selective monoamine reuptake inhibitors (MARI): Desipramine, Imipramine, Clomipramine, Amitriptyline, Nortriptyline, Protriptyline, Doxepin, Trimipramine  Monoamine oxidase inhibitors, non-selective (MAOI): Phenelzine, Tranylcypromine  Other: Hydroxytryptophan, Mianserin, Trazodone, Nefazodone, Mirtazapine*, Bupropion, Vilazodone |
| Antipsychotics | First-generation: Perazine, Periciazine, Pipotiazine, Haloperidol*, Bromperidol, Benperidol, Droperidol*, Molindone, Flupentixol, Clopenthixol, Chlorprothixene, Thiothixene, Zuclophenthixol, Fluspirilene, Pimozide, Penfluridol, Loxapine, Clotiapine,  Second-generation: Melperone, Sertindole, Ziprasidone, Lurasidone, Clozapine, Olanzapine*, Quetiapine*, Asenapine, Remoxipride, Amisulpride, Risperidone, Aripiprazole, Paliperidone, Iloperidone, Cariprazine, Amoxapine, Sulpride, Sultopride, Veralipride, Prothipendul, Mosapramine  Lithium |
| Anxiolytics | Anxiolytic: Meprobamate, Buspirone, Secobarbital, Chloral Hydrate, Ramelteon, Scopolamine  Anxiolytic and antiepileptic: Phenobarbital  Benzodiazepine: Tetrazopan, Clonazepam, Diazepam, Chlordiazepoxide, Medazepam, Oxazepam, Clorazepate, Lorazepam*, Adinazolam, Bromazepam, Clobazam, Ketazolam, Prazepam, Alprazolam, Halazepam, Pinazepam, Camazepam, Nordazepam, Clotiazepam, Cloxazolam, Bentazepam, Flurazepam, Nitrazepam, Estazolam, Triazolam, Lormetazepam, Temazepam, Midazolam, Brotizolam, Quazepam, Loprazolam, Cinolazepam  Benzo-like: Zopiclone, Zolpidem, Zaleplon, Eszopiclone, Etizolam |

Note: medications marked with an * can also be used off-label to treat pregnancy-related nausea
